# Low Altitude Solar Magnetic Reconnection, Type III Solar Radio Bursts, and X-ray Emissions

**DOI:** 10.1038/s41598-018-19195-3

**Published:** 2018-01-26

**Authors:** I. H. Cairns, V. V. Lobzin, A. Donea, S. J. Tingay, P. I. McCauley, D. Oberoi, R. T. Duffin, M. J. Reiner, N. Hurley-Walker, N. A. Kudryavtseva, D. B. Melrose, J. C. Harding, G. Bernardi, J. D. Bowman, R. J. Cappallo, B. E. Corey, A. Deshpande, D. Emrich, R. Goeke, B. J. Hazelton, M. Johnston-Hollitt, D. L. Kaplan, J. C. Kasper, E. Kratzenberg, C. J. Lonsdale, M. J. Lynch, S. R. McWhirter, D. A. Mitchell, M. F. Morales, E. Morgan, S. M. Ord, T. Prabu, A. Roshi, N. Udaya Shankar, K. S. Srivani, R. Subrahmanyan, R. B. Wayth, M. Waterson, R. L. Webster, A. R. Whitney, A. Williams, C. L. Williams

**Affiliations:** 1 School of Physics, University of Sydney, Sydney, NSW 2006 Australia; 2 Centre for Astrophysics, School of Mathematical Sciences, Monash University, Melbourne, VIC 3800 Australia; 3 International Centre for Radio Astronomy Research, Curtin University, Perth, WA 6845 Australia; 4 National Centre for Radio Astrophysics, Tata Institute for Fundamental Research, Ganeshkhind, Pune, 411007 India; 5 Department of Physics, Seattle University, Seattle, WA 98122-1090 USA; 6 The Catholic University of America, Washington, DC USA; 7 NASA Goddard Space Flight Center, Greenbelt, MD 02330 USA; 8 Department of Cybernetics, Tallinn University of Technology, Tallinn, 12 618 Estonia; 9 Square Kilometre Array South Africa (SKA SA), Cape Town, South Africa; 10 Harvard-Smithsonian Center for Astrophysics, Cambridge, USA; 11 Rhodes University, Grahamstown, South Africa; 12 Arizona State University, Tempe, USA; 13 MIT Haystack Observatory, Westford, MA 01886-1299 USA; 14 Raman Research Institute, Bangalore, India; 15 MIT Kavli Institute for Astrophysics and Space Research, Cambridge, USA; 16 University of Washington, Seattle, USA; 17 Victoria University of Wellington, Wellington, New Zealand; 18 University of Wisconsin–Milwaukee, Milwaukee, USA; 19 University of Melbourne, Melbourne, Australia; 20 National Radio Astronomy Observatory (NRAO), Charlottesville, USA; 21 ARC Centre of Excellence for All-sky Astrophysics (CAASTRO), Sydney, USA; 22 Australian National University, Canberra, Australia; 23 Present Address: Space Weather Services, Bureau of Meteorology, PO Box 1386, Sydney, NSW 1240 Australia

## Abstract

Type III solar radio bursts are the Sun’s most intense and frequent nonthermal radio emissions. They involve two critical problems in astrophysics, plasma physics, and space physics: how collective processes produce nonthermal radiation and how magnetic reconnection occurs and changes magnetic energy into kinetic energy. Here magnetic reconnection events are identified definitively in Solar Dynamics Observatory UV-EUV data, with strong upward and downward pairs of jets, current sheets, and cusp-like geometries on top of time-varying magnetic loops, and strong outflows along pairs of open magnetic field lines. Type III bursts imaged by the Murchison Widefield Array and detected by the Learmonth radiospectrograph and STEREO B spacecraft are demonstrated to be in very good temporal and spatial coincidence with specific reconnection events and with bursts of X-rays detected by the RHESSI spacecraft. The reconnection sites are low, near heights of 5–10 Mm. These images and event timings provide the long-desired direct evidence that semi-relativistic electrons energized in magnetic reconnection regions produce type III radio bursts. Not all the observed reconnection events produce X-ray events or coronal or interplanetary type III bursts; thus different special conditions exist for electrons leaving reconnection regions to produce observable radio, EUV, UV, and X-ray bursts.

## Introduction

Satellites and ground instruments often observe intense radio waves and X-rays. Their sources include the Sun, Jupiter, supernovae, pulsars, the galactic centre, and active galactic nuclei, as well as lightning flashes, the auroral regions, and magnetosphere of the Earth. Most X-rays are produced when energetic electrons collide with other particles in the plasma (ionised gas). Similar single-particle processes produce some radio emissions (e.g., synchrotron emission from supernovae) but are relatively weak. The brightest radio sources (e.g., the Sun and pulsars) involve collective processes, in which energised plasma particles interact collectively to generate intense plasma waves and radio emission. Magnetic reconnection, in which magnetic energy is converted into directed particle kinetic energy and heated plasma, is very attractive as both the energy release mechanism and the source of energetic particles producing radio and X-ray bursts. Multiple critical problems remain unsolved in astronomy, space physics, and plasma physics concerning how magnetic reconnection and collective radio emissions occur.

Type III solar radio bursts^1–4^ are the Sun’s most intense and frequent transient nonthermal emissions and are one of four known archetypes for collective nonthermal radio emission from plasmas^5^. Type III bursts (often abbreviated below as “type IIIs”) are produced near the electron plasma frequency f_p_ and near 2f_p_ by linear and nonlinear processes involving Langmuir waves generated near f_p_ by a fast beam of electrons with speeds ≈ (0.1–0.7)c^1–6^. (Here c is the speed of light and in SI units f_p_ ≈ 9 n_e_^1/2^ Hz, where n_e_ is the ambient plasma’s electron number density.) Spacecraft in the solar wind routinely detect the fast electrons, Langmuir waves, and radio emission^7–12^. Type IIIs drift rapidly from frequencies of order 200 MHz to 20 MHz in a few to 30 seconds and from 20 MHz to 20 kHz in an hour, as the electrons move from radial distances below 1.2 solar radii (R_S_) from the Sun’s centre to several R_S_ and then through the interplanetary medium (solar wind) to 1 AU (215 R_S_), respectively. For over 50 years the source of type III electrons has been believed but not proven to be solar magnetic reconnection regions^2,3,13–16^. Electrons are energized there, either by electric fields parallel to the magnetic field or by wave-particle interactions, and develop a localized beam or bump in velocity space due to faster electrons outrunning slower electrons from a transient release, often called “time-of-flight” effects.

**Figure 1:**
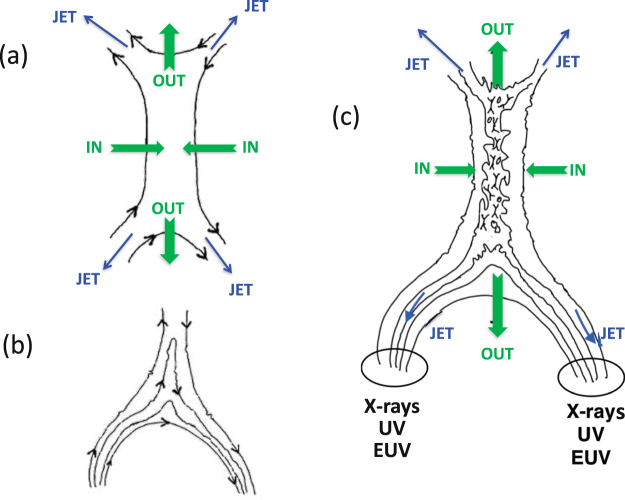
(**a**) Standard cartoon of a microscopic magnetic reconnection site, showing the inflowing and outflowing plasma and magnetic field lines plus the 4 “jets” of energized and heated particles close to the magnetic separatrices. (**b**) A “cusp”current sheet near the apex of a macroscopic magnetic field loop. (**c**) Turbulence, 3D, and other effects in a realistic coronal loop – cusp structure lead to a macroscopic current sheet with multiple embedded microscopic reconnection regions that combine to form macroscopic outflows and 4 macroscopic jets; the downgoing ones cause standard optical flares and UV, EUV, and X-ray emissions near the magnetic footpoints.

Magnetic reconnection is a fundamental but relatively poorly observed and understood process for the conversion of magnetic field energy into plasma motion, energized/heated plasma, and changing magnetic topologies^13,14^. It is considered to be important in multiple laboratory, space, and astrophysical contexts, including solar flares^13–16^, coronal mass ejections^17^ and transient radio and X-ray^2,3,18–21^ events. The standard microscopic picture involves two plasma regions with antiparallel magnetic fields ±**B** moving together (Fig. 1a), resulting in a current sheet along their joint boundary with thickness between the electron and ion gyroradii. Large currents and electric fields exist in the current sheet. When suitable anomalous resistivity and inflows occur, the magnetic field lines are reconnected into a different topology and expelled with the plasma almost along the current sheet at speeds close to the Alfven speed. Heated and accelerated particles also escape along magnetic field lines close to the boundary between the reconnected and original field lines. Thus, the plasma outflow and energetic particles are expected to form two pairs of “jets”, with one jet on each side of each end of the current sheet (Fig. 1a). These jets can be close to parallel, as found here for the outgoing jets, or widely diverging, as found here for downgoing jets that connect to well-separated loop footpoints. Mechanisms for energizing and heating the particles are discussed elsewhere^22,23^.

Consider a solar loop with a “cusp” current sheet near the apex (Fig. 1b), where the magnetic field strength is weakest. Plasmas are naturally turbulent over a wide range of macroscopic scales down to the ion gyroradius (and below), corresponding to local changes in the direction and magnitude of **B** plus other plasma parameters. Twisting and motion of the magnetic field lines near the current sheet, due to turbulence and external forces, then cause the anti-parallel field lines in the current sheet to move together, leading to magnetic reconnection. This plausibly develops in a macroscopic and observable volume for several reasons: folding and twisting of the current sheet in 3D by turbulence causes multiple regions with anti-parallel **B** (and so reconnection) to develop^24–27^; the current sheet is unstable along its axis to so-called tearing instabilities; these two effects plus any “guide” magnetic field (into the page) break translational symmetry along the line of sight and dramatically broaden the current sheet^27^; and projection effects when the 3D loop-cusp structure is viewed at a significant angle to the loop’s symmetry plane. Thus, reconnection in a realistic coronal loop – cusp structure is expected to develop along the lines in Fig. 1c, with a macroscopic current sheet that has multiple embedded microscopic reconnection regions (Fig. 1a) with outflows. Although fine structures are likely, the combination of these individual regions should lead to two bulk reconnection outflows, one Sunwards and one outwards, and to two pairs of jets of energetic particles, one pair Sunwards towards two magnetic footpoints and one pair outwards along open field lines (Fig. 1c). These pairs of macroscopic jets of energetic particles are required by the macroscopic symmetries and connections of the magnetic field: each downgoing jet goes to one magnetic footpoint, while two separate outgoing jets develop due to the cusp geometry having oppositely directed **B** fields on each side of the cusp at large distances.

Pairs of jets have not yet been observed. Yohkoh and Hinode X-ray images^15,16,28–31^ sometimes show large-scale X-ray jets and magnetic field configurations that are interpreted in terms of reconnection, bulk plasma motion (both outwards and Sunwards), and heating in or near the putative reconnection region. However, neither these nor recent published Solar Dynamics Observatory (SDO)^31–33^, TRACE^34^, or Hi-C^35^ images of reconnection spatially resolve the predicted pair structures and only a few discuss radio events^31,33,34^. Data from RHESSI and earlier satellites show bursts of X-rays produced near the tops and footpoints of magnetic loops, the latter when the energetic electrons and bulk outflow particles collide with chromospheric plasma and produce bremsstrahlung X-rays^14–21,28–31,33,35^. Whether the electrons producing the radio and X-ray emissions are the same or different remains unknown^18–20,36,37^, with further acceleration by parallel electric fields and/or wave-particle interactions possible^36^, similar to Earth’s auroral region.

Rapid observing cadences and simultaneous multi-wavelength imaging are necessary to go beyond statistical arguments and convincingly demonstrate causal connections between specific type III radio bursts, magnetic reconnection events, and X-ray bursts and thereby test the standard model for type III bursts. For instance, although an important step, finding a 60% time correlation on a minute timescale between EUV jets indicative of reconnection and interplanetary type III bursts (frequencies ~ 5 MHz and heights ~ 5 R_S_)^31^ is inadequate since type IIIs have a lifetime ~ 1–10 s near the electron source (f ~ 50–500 MHz) and there were no radio imaging or X-ray observations to demonstrate physical connections. Similarly, a recent important analysis of fragmentary decimetric type III bursts (type IIIdm) for 1–2 GHz (heights < 0.1 R_S_) without coronal and interplanetary extensions does show good radio – X-ray correlations in flux (over a factor of only ~ 2 in magnitude) and position^33^; however, the EUV event has a much larger duration than expected (7 minutes versus several ~ 20 s intervals), does not have the pair structure, cusp features, and macroscopic outflows expected for reconnection events, and detailed EUV – radio – X-ray correlations are not presented. Similar issues arise for weak fragmentary type IIIs in the range 250–600 MHz (without interplanetary extensions) for a limb event^34^ with X-ray sources at magnetic footpoints and multiple EUV jets that develop close to the footpoints and plausibly extend to the imaged radio sources. Thus, while a qualitative connection is widely accepted between X-ray events, type IIIs, and reconnection^15,16,18–35^, the detailed connections between type IIIs, reconnection events, solar flares, and X-ray events still need to be established.

Here we present definitive evidence for: first, solar magnetic reconnection events with double-sided jets, current sheets and cusp-like geometries on top of loops, plus strong outflow along pairs of open magnetic field lines; and, second, direct causal links between reconnection events and both large type III events (coronal and/or interplanetary) and X-ray bursts, with simultaneity in both time and spatial position. We also demonstrate the occurrence of other reconnection and X-ray events without well-correlated large type IIIs, as well as weak radio emissions at other times; these are interpreted as evidence for special conditions being necessary for the production of observable type III and X-ray emission by reconnection events. Novel data are used, including EUV-UV images from the Atmospheric Imaging Assembly (AIA)^38,39^ and EUVI instruments on NASA’s Solar Dynamics Observatory (SDO) and STEREO B spacecraft, respectively, AIA movies, radio dynamic spectra and images from the Murchison Widefield Array (MWA)^40–43^, radio dynamic spectra from the Learmonth radio spectrograph and STEREO B, and X-ray fluxes and images from NASA’s RHESSI spacecraft^44^.

## Results

This paper addresses events during the period 01:05–01:30 UT on 25 September 2011. They originated in active region NOAA AR11302 (referred to without the prefix NOAA below), which had at least 3 large sunspots and was then located near the Sun’s east equatorial limb as viewed from Earth. Some of the events were associated with very weak but detectable X-ray flares observed by the GOES spacecraft near 01:11:50, 01:13:20, and 01:19:50 UT, but not with C or larger class X-ray flares once the background is subtracted. (See Supporting Material A for more detail).

### Evidence for magnetic reconnection

**Figure 2:**
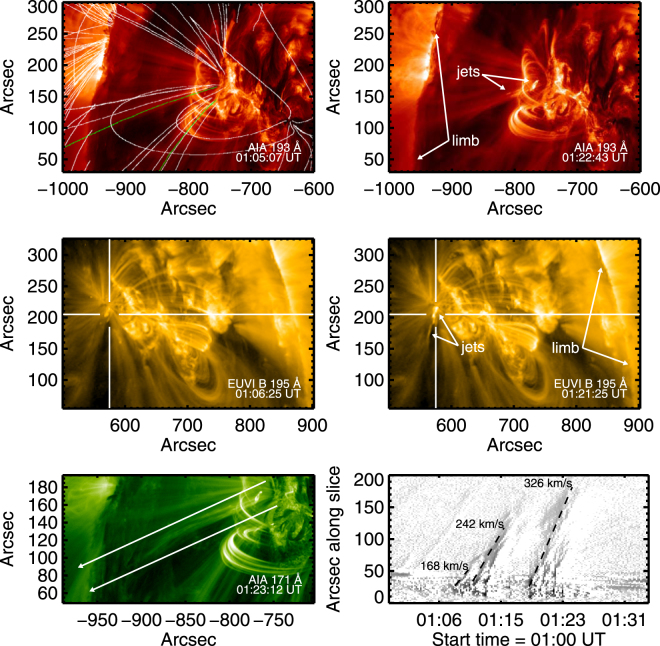
Solar images near AR11302 on 25 September 2011: Largescale images from (top row) SDO-AIA at 193 Å and (middle row) STEREO B’s EUVI instrument at 195 Å, showing the locations of the active region and its magnetic loops, solar limb, open magnetic field lines, and associated jets and outflows during a quiet period (≈01:05 UT) and during a reconnection event (≈01:22 UT). The reconnection region is centered near X = −770 and Y = 170 arcsec. The SDO viewpoint is from Earth (top panels) while the STEREO-B viewpoint (middle panels) is from near 120 degrees eastwards of the Sun-Earth line, corresponding to clockwise from Earth along Earth’s orbit. Magnetic field lines obtained from the PFSS model are overplotted on the top-left panel, with green and white indicating open and closed field lines, respectively. Cross-hairs in the middle panels identify the reconnection region and downward flows, viewed from STEREO B. The bottom row shows a zoomed-in 193 Å SDO-AIA image (left), with outflows along open magnetic field lines bounded approximately by the white lines, and a position-time plot of the outflows (right), formed by summing the intensity transverse to the white lines as a function of distance along the white lines. North, south, east, and west are the top, bottom, left, and right sides of the images. See text body for further details and online materials for corresponding movies.

**Figure 3:**
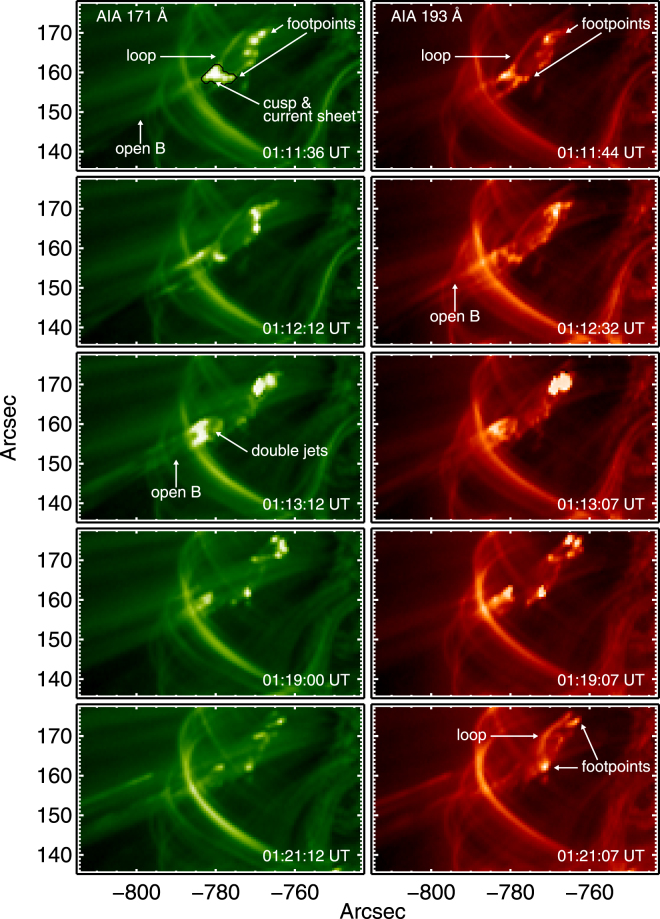
Strongly zoomed-in SDO-AIA images of reconnection regions near AR11302 on 25 September 2011 at 171 Å (left) and 193 Å (right): double-sided upward-directed jets, downward-directed jets, current sheets and cusp-like geometries on top of low-lying magnetic loops are clearly visible, as well as projected transverse-directed higher loops. The black contour in the top-left panel bounds the region used for the DEM calculation. See text body for further details and online materials for corresponding movies.

SDO data from the AIA instrument^38,39^ provide direct evidence for magnetic reconnection occurring in AR11302 during this period. The evidence is in the form of images of enhanced 93–1700 Å EUV emissions with pairs of upward- and downward-directed jets, current sheets and cusp-like geometries on top of low-lying magnetic loops, and strong (upward) outflow events along open magnetic field lines that occur in pairs and are magnetically connected to the jets and cusp-like regions (Figs 2 and 3 plus, especially, Movies B1, B2 and B3 of Supporting Material B). These are the idealized signatures and geometry expected for magnetic reconnection in a symmetric current sheet at the top or side of a magnetic loop. Note that while the outflows and fast particles from reconnection regions should produce four jets in principle (a pair along ±**B** for each outflow direction), plasma inhomogeneities and spatial asymmetries may lead to jets being observably different and even unobservable.

In more detail, Fig. 2 shows snapshots of the large-scale environment and medium zooms of the 193 Å and 171 Å SDO-AIA data and 195 Å STEREO B–EUVI data for AR11302, Fig. 3 shows zoomed-in images for 171 Å and 193 Å SDO-AIA data, and Movies B1 and B2 show large-scale and zoomed-in 171 Å data. The top and middle pairs in Fig. 2 show views from the Sun-Earth direction (SDO) and STEREO B (near 120 degrees clockwise from Earth along Earth’s orbit), respectively, before and during reconnection events. The upper-left panel of Fig. 2 includes a potential field source surface (PFSS^45^) prediction for the coronal magnetic field near 1230 UT (see Method section). The PFSS predictions show good agreement with magnetic structures observed in the AIA data, including open B lines in the jet region and closed loops at low altitude. Noting the locations of the solar limb in these four images, the brightenings and activity are associated with open magnetic field lines and low-lying loops that are behind and southeast (viewed from Earth towards STEREO B) and well below the multiple-loop region in the foreground of the SDO images. Zoom images in Figs 2 and 3 and Movies B1 and B2 show jets that are connected to outflows along the open magnetic field lines (Movie B3), as well as brightenings at the footpoints of the low-lying loops. The strongly zoomed-in SDO images in Fig. 3 and Movie B2 demonstrate the existence of one or more long-lived time-varying current sheets with cusp-like geometries above the low-lying loops (near 01:11:40, 01:12:20, 01:19:00, and 01:21:10 UT particularly) and of connected transient double-sided jets oriented approximately southeast-northwest with paired structure in the southeast- and outwards-directed jets (near 01:11:40, 01:13:10, and 01:19:00 UT particularly), the latter partly obscured by overlying loops. The looping, forward-backward, nature of Movie B2 is particularly useful in showing pair structure in the downward-directed jets, which diverge widely and show clear downwards motion away from the reconnection site along two bundles of magnetic field lines connected to distinct foot points, one far and one apparently close to the reconnection site. Movie B2 also shows pairs of upward jets, plus activity on an even lower-lying magnetic structure. STEREO B data show pair structure for downward-directed jets going to distinct footpoints and show upward jets without clear pair structure, presumably due to the different viewing geometries and instrument resolutions and cadences.

The heights of the loops and current sheets of the reconnection regions are low, estimated to be < 5–10 Mm or 10^−2^ R_S_ (solar radius) from a semi-circular approximation and the distances measured directly from Fig. 3 and Supporting Material B. The loops appear to be on their sides, oriented approximately southeast and not radially, with shorter distances from the current sheet to the more southeasterly footpoints than to the northwesterly footpoints. Analyses of other events yield larger heights: 15–30 Mm^31^, 15 Mm^33^, and 10–20 Mm^34^. Heights < 10 Mm correspond to the nominal chromosphere.

The electron temperature and density averaged over the cusp region are available using a differential emission measure (DEM) analysis described in the Methods section: the average, DEM-weighted, values are T_e_ = 6.3 MK and n_e_ ≈ 2.3 × 10^10^ cm^−3^. The cusp thus has a moderate flare temperature and is significantly hotter than the ambient corona (1 MK), consistent with reconnection occurring in the current sheet and heating the plasma.

The zoomed-in and large-scale SDO images in Figs 2 and 3 and Movies B1, B2, and B3 demonstrate the existence of strong transient outflows along pairs (typically) of open magnetic field lines that connect to the time-varying jets and cusp regions and that agree well with the PFSS model. Examples occur near 01:13:00–01:14:20, 01:16:00–01:17:20, 01:20:19–01:21:31, and 01:22:00–01:23.53 UT. Outflow speeds are estimated by defining a spatial domain containing the open field lines (white lines in Fig. 2’s bottom-left panel and the white box in Movie B3’s bottom panel) and measuring the position of peaks in the intensity as a function of position and time (Methods section). Ignoring projection effects, outflow speeds ≈ 170–330 km s^−1^ are found for 3 prominent events (Fig. 2’s bottom-right panel). These outflows are fast enough to be relevant to the solar wind’s origin and acceleration but are a factor 2–4 slower than the expected solar wind speeds beyond 10 R_S_. Since magnetic reconnection produces bulk outflows slower than the Alfven speed V_A_^14–18^, the observed outflows imply V_A_ ≥ 170 km s^−1^. Combining the PFSS model’s value B ≈ 70 G in the cusp with an average electron number density n_e_ ≈ 2.6 × 10^10^ cm^−3^ from the DEM analysis (Methods section) yields V_A_ ≈ 1000 ± 700 km s^−1^ in the cusp. This estimate is consistent with the outflows corresponding to reconnection, albeit without realistic uncertainty estimates and with lower V_A_ expected further along the current sheet. The corresponding electron beta, the ratio of thermal to magnetic energy, is β_e_ ≈ 0.10. Note that the bulk outflowing plasma is not responsible for the radio emission events described next.

### Type III radio bursts

**Figure 4:**
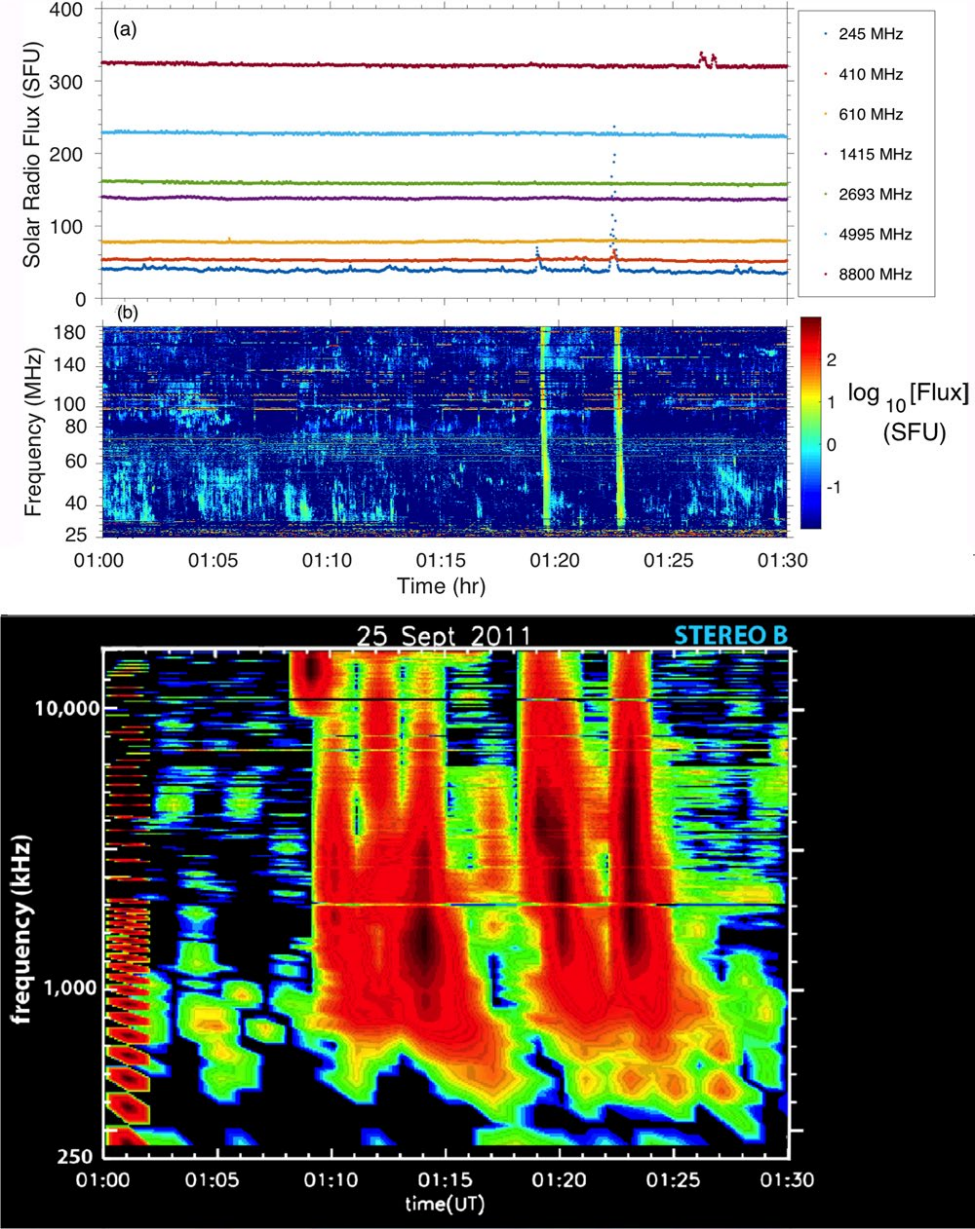
Radio data for the period 01:00–01:30 UT on 25 September 2011: (**a**) Learmonth RSTN fluxes as a function of frequency and time, showing activity at 245 and 410 MHz near 01:19 and 01:12 UT; (**b**) calibrated dynamic spectrum over the 25–180 MHz range of the Learmonth radio spectrograph, showing outbursts near 01:19 and 01:22 UT of multiple type III events; (**c**) Radio dynamic spectrum from NASA’s STEREO B spacecraft for frequencies 250 kHz–12 MHz, with intensity colour-coded (the intensity decreases from red to yellow, green, blue, and black). The rapidly-drifting, almost vertical, signals near 01:10, 01:12, 01:14, 01:19, and 01:23 UT are weak interplanetary type III bursts.

**Figure 5:**
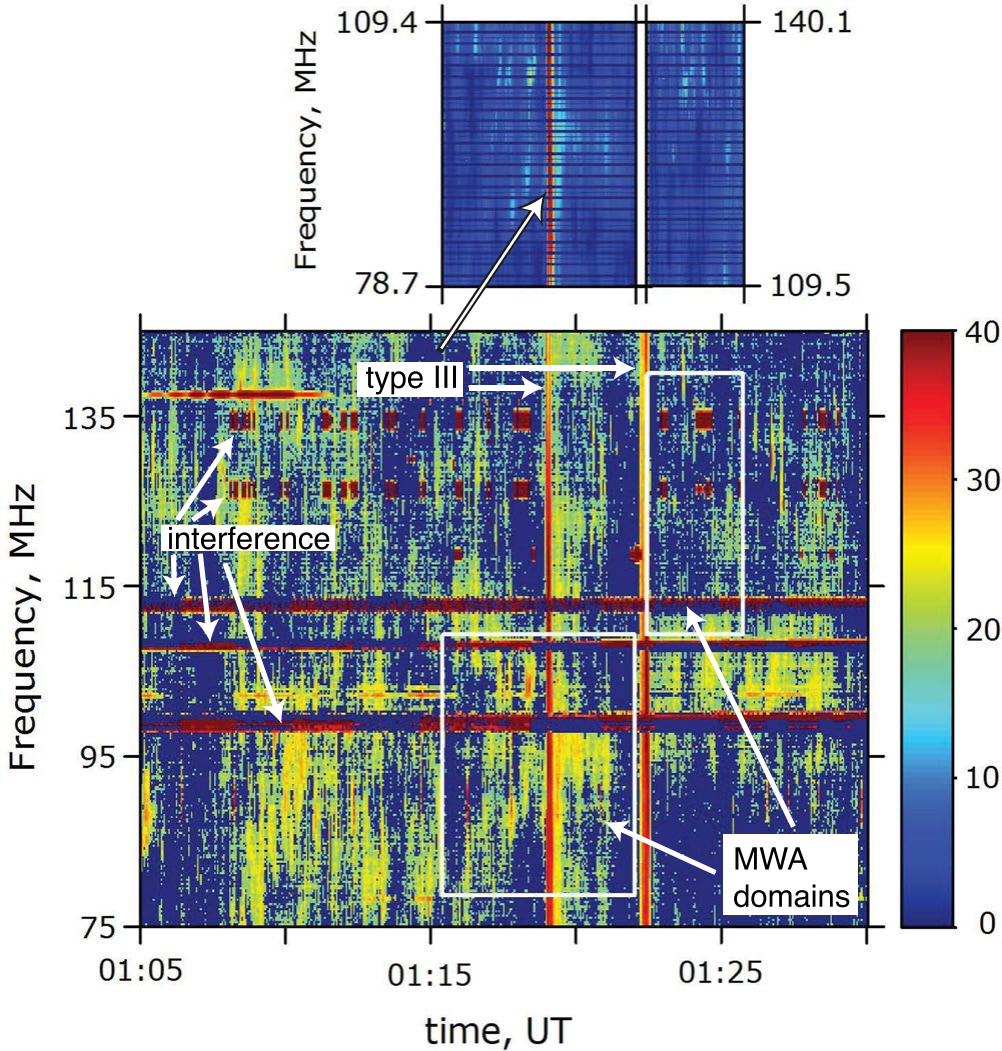
Radio observations for 01:05–01:30 UT on 25 September 2011: (top) the 32-tile prototype MWA for the domains 78.7–109.4 MHz and 109.5–140.1 MHz and (bottom) the Learmonth radiospectrograph for 75–140 MHz, with the MWA domains marked using white boxes. Frequency-dependent instrumental backgrounds were subtracted from both datasets. Amplitude scale is in dB from the background. Strong type IIIs are visible across the full frequency domains near 01:19 and 01:22 UT. Strong intermittent interference causes horizontal bands in the Learmonth data (e.g., near 98–99 and 125 MHz).

Strong coronal type III radio events occurred in two outbursts, 01:18:50–01:19:20 and 01:22:00–01:22:35 UT, from approximately 410 MHz to below 25 MHz, as shown in Fig. 4(a) by detections at 410 and 245 MHz by the Radio Solar Telescope Network (RSTN) station at Learmonth and over the entire frequency range 25–180 MHz of the Learmonth radiospectrograph in Figs 4(b) and 5. These are classic type III bursts for outward moving electrons, as evidenced by the duration of each group of bursts increasing with decreasing frequency in Learmonth data and their extension in Fig. 4(c) into the interplanetary domain. The type IIIs agree very well in time with the major EUV/reconnection events, as demonstrated below.

Removal of Learmonth’s instrumental backgrounds and some interfering signals reveals (Fig. 5, bottom panel) that the 01:19 and 01:22 UT outbreaks of intense type IIIs are superposed on multiple weak emissions that are localized in frequency f and time t. Data from the prototype of the MWA^40–43^ (1/4 of the dipoles of the full MWA and poorer angular resolution) confirm both the 01:19 and 01:22 type III outbursts and the weak events (Fig. 5, top panel), although the 01:22 event mostly occurred during a change in observing mode. These weak events may be weak type III bursts that are above instrumental backgrounds only in limited f - t domains^46–50^ or short duration and small bandwidth events first detected by MWA^40^ and recently interpreted as evidence for localized coronal heating events^49^.

The 01:19 and 01:22 UT groups of metric type III bursts observed by Learmonth and MWA extend into the interplanetary domain and are clearly detected by the STEREO B spacecraft in the 0.5–16 MHz domain, as shown in Fig. 4(c). STEREO A detects neither event (not shown). Evidence that the STEREO B events are extensions of the 01:19 and 01:12 metric type IIIs includes the timing of the radio and EUV events, the locations of STEREO A and B off the Sun’s west and east limbs, respectively, and the reconnection events in Figs 2 and 3 being near the Sun’s east limb (so that STEREO B is favoured to detect the radio emission). A data gap 01:00–02:00 UT for the radio instrument on the Wind spacecraft, located near the Sun-Earth line, precludes checking these interpretations and arguments using Wind data. In addition, STEREO B data (but not STEREO A data) show 3–4 interplanetary type IIIs that peak in the range 10–16 MHz near 01:09:30, 01:10:30 (these last two may be the same event), 01:12:30, and 01:14:30, being between 15 and 40 dB above background. Again, STEREO A detects none of these events, thus supporting an eastern source. Interplanetary type IIIs without significant metric type III emission thus occur in association with many of the SDO/AIA outbursts.

### Radio, X-ray, and EUV/UV source regions

**Figure 6:**
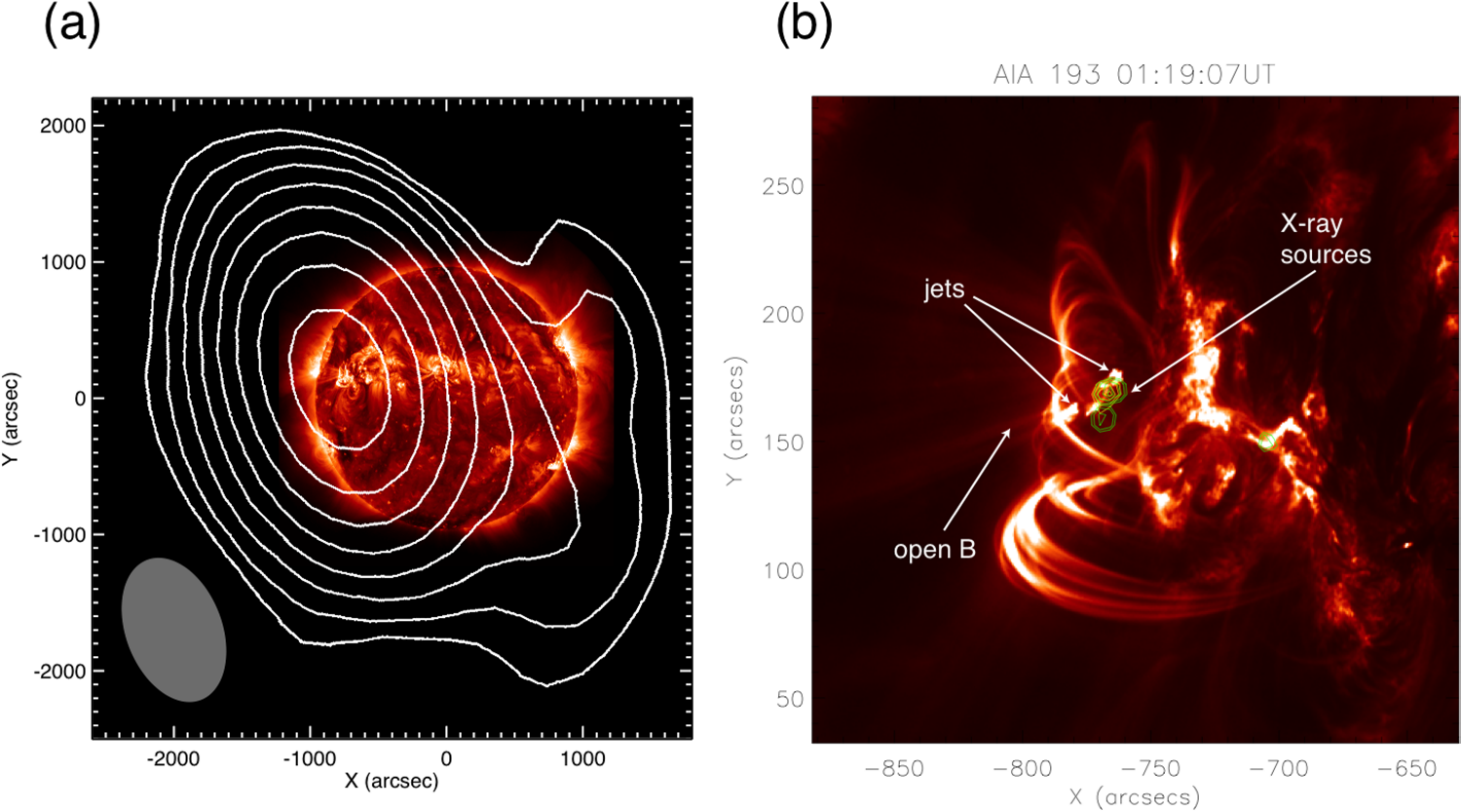
Source locations of (**a**) MWA radio source at 01:22 UT and 138 MHz (black contours) superposed on a SDO AIA 193 Å image from 0113 UT and with the synthesized MWA beam shown (ellipsoid in the lower left) – these are aligned by comparing the time- and frequency-varying radio sources over the solar disk for the approximate period 01:00–03:00 UT (including sources near both the east and west limbs) with the astrometrically precise SDO image, then rotated into solar coordinates with solar north vertically up – and (**b**) RHESSI sources at 3–6 keV (green contours) and 12–25 keV (yellow contours) at 01:19:07 superposed onto the SDO-AIA image at 193 Å for time 01:19:07 UT.

The first MWA image of a type III source region, Fig. 6(a) superposes MWA’s 0122 UT radio source at 138 MHz (in a single 40 kHz channel for a 1-s snapshot) onto the SDO satellite’s AIA image at 304 Å from 01:13 UT. The radio source for this time and frequency is consistent with the spatial locations of AR11302, the SDO EUV events in Figs 1 and 2, and the radio results for other frequencies and times during this event. A Gaussian fit to the radio intensity image gives a radio source size of <15″, much less than half a solar radius. Images near 01:19 UT yield similar results but with worse angular resolution since f < 107 MHz for those MWA observations. (The full MWA has improved spatial resolution and sensitivity, recently exploited to interpret novel type III dynamics in terms of diverging magnetic fields for an event also associated with EUV jets^43^).

**Figure 7:**
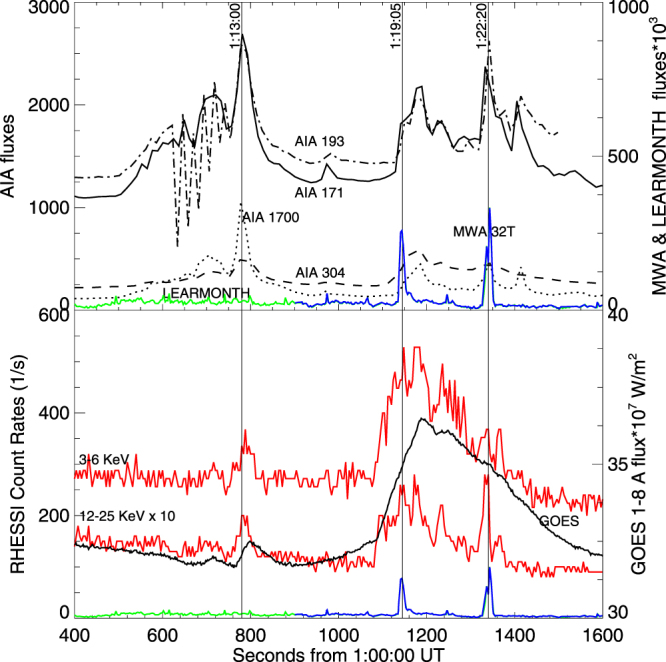
Energy fluxes versus time for the period 01:06:40–01:26:40 UT: SDO-AIA wavelengths 171, 193, 304, and 1700 Å, masked over the spatial domain of interest, MWA (blue line) and Learmonth (green line) integrated over their frequency domains (arbitrary units), RHESSI 3–6 and 12–25 keV (red curves), masked over the spatial domain of interest, and GOES 1–8 Å flux.

Figure 6(b) compares the SDO 193 Å image for time 01:19:07 UT with the source locations of impulsive X-rays in the bands 6–12 keV and 6–25 keV measured by RHESSI^44^. The regions of high 6–25 keV intensity coincide with SDO’s northeast-directed (downgoing) reconnection jet, consistent with electrons from the reconnection region producing the X-rays. These regions appear first in this image. Similar results are found for the 01:22 UT event. In contrast, the weak source near (X,Y) ≈ (−700″,150″) in Fig. 7(b) started near 01:18:00 UT and disappeared shortly after 01:19:07 UT. It appears unrelated to the SDO reconnection jets and the type IIIs.

### Temporal and causal relationships between the EUV, radio, and X-ray events

Close temporal connections are demonstrated quantitatively in Fig. 7 between the (metric) radio, EUV, and X-ray emissions. These “light curves” show unequivocally that the strong type III bursts are associated with major impulsive increases in EUV and UV emission (from 93 to 1700 Å) and also with impulsive increases in 3–6 keV and 12–25 keV X-rays. STEREO data show these type IIIs continuing into the interplanetary medium to near 0.5 MHz. (The 3–25 keV onset near 01:18:00 UT is due to the weak source near (X,Y) ≈ (−700″,150″) in Fig. 6(b), whereas the peak near 01:19:05 UT and following activity is associated with the SDO reconnection jets). Thus, within the instrumental uncertainties, the radio, UV, EUV, and impulsive X-ray sources are temporally and spatially coincident. All the expected characteristics of magnetic reconnection are observed in the SDO images and the RHESSI X-ray data. Accordingly these new data and analyses provide the first direct evidence that the electron beams responsible for strong type III bursts flow away from and are produced in magnetic reconnection regions. This is the long-sought evidence for the standard model of type III bursts.

The SDO, Learmonth, MWA, RHESSI, STEREO, and GOES data in Figs 2–7 show that not all reconnection events or distinct bursts of enhanced EUV, UV, and impulsive X-ray emissions are associated with strong coronal and interplanetary type III bursts. First, the strong SDO, RHESSI, and GOES events near 01:12:30–01:14:00 UT are associated with either no metric radio events or at best very weak bursts localized in frequency and time, although the STEREO B radio event in the 10–16 MHz range near 01:13:30–01:15:00 UT is plausibly associated. Second, the strong, multiply-peaked EUV-UV emissions 01:08–01:13 UT are associated with neither enhanced X-rays nor metric radio bursts, but are associated with the 3–4 weak interplanetary type IIIs detected by STEREO B 01:08–01:14 UT (Fig. 4). Third, although the onsets and peaks in the EUV/UV emissions have clear counterparts in the X-rays for the period 01:19–01:23 UT (recognizing that X-ray profiles are much more spiky and are contaminated 01:18:10–01:19:10 UT by Fig. 6(b)’s confusion source), this is not true for the periods 01:08–01:13 and 01:23–01:24 UT. Indeed, many more X-ray and radio events occur after 01:15 UT than EUV/UV events, whereas the converse is true before 01:15 UT. Finally, Fig. 4’s metric radio data show multiple weak bursts localized in frequency and time for the period 01:05–01:30 UT that are not associated with enhanced EUV or X-ray emissions and occur on much shorter time scales than the observed X-ray and UV activity.

While EUV, UV, X-ray and radio activity may occur on faster timescales than the instrumental cadences, the four points above suggest that, even for causally-connected events, special conditions are required to produce observable radio and X-ray emission from electrons leaving reconnection regions and producing enhanced EUV and UV emission. Thus, Fig. 7 provides strong evidence that the imperfect correlations observed previously between X-ray, radio, EUV, and magnetic reconnection events^18–20,31–35^ are intrinsic (due to different physical conditions being required to produce observable signatures) and not due to there being multiple simultaneous but uncorrelated sources active across the Sun (e.g., the two X-ray sources in Fig. 6(b)) or to instrumental limitations. The imperfect correlation demonstrated above between metric and interplanetary type IIIs, and the fact that these emissions are often observable only in limited frequency-time domains, are also evidence that different special conditions are required to have observable radio.

## Discussion and Conclusions

One interpretation for these special conditions is that two different (but related) electron populations originating in reconnection regions produce type III bursts and X-ray bursts in distinct source regions. Specifically, an auroral-style acceleration process, involving parallel electric fields and Alfven waves as in Earth’s aurora, also acts on the downgoing electrons as they near the magnetic footpoints, but not on the outward-going electrons on open field lines that produce type IIIs^20,32^ (Supporting Material C). Adding this acceleration process may also resolve the so-called “number problem”^20,36,37^, which is that too many accelerated electrons are required to explain the observed X-rays in terms of thick-target bremstrahlung from electrons accelerated in coronal reconnection regions, since the radio and X-ray emitting electrons would then not be produced by a single acceleration process in the same region. The low (~5–10 Mm) chromospheric heights of this paper’s reconnection regions imply a much denser plasma than the corona and so also reduce the number problem.

Another interpretation involves the fundamentally different physics of incoherent single-particle emission processes versus coherent collective emission processes, only the latter involving amplification due to individual particles emitting together. Specifically, the X-ray emissions are primarily bremstrahlung by individual electrons and the EUV emissions are primarily due to single-particle collisions of energetic particles with atoms and ions that lead to enhanced atomic and ionic line emission, while type III bursts involve collective processes for the growth of Langmuir waves and their conversion into radio emission^4–12,48,50^. A priori then, the conditions for observable emissions should be different. Detailed physical arguments also exist (Supporting Material C) that more intense type III emission is favoured for more rapid release events in smaller regions, balanced by the need for the source region to be large enough for the emission to be observable, while a larger acceleration region will produce more fast electrons and so X-ray and EUV emission than a smaller source for otherwise identical electron and source conditions. Thus, the different growth physics (especially collective versus single-particle) and dependences on release time and size of the acceleration region make it very plausible that different special conditions exist for production of observable radio, X-ray, and EUV emission from energetic electrons leaving solar magnetic reconnection regions.

In conclusion, this paper provides direct evidence for magnetic reconnection events in new SDO observations of enhanced EUV/UV events, including double-sided jets, cusp-like geometries on top of loops, and strong outflow along open magnetic field lines. MWA and Learmonth data show strong type III solar radio bursts in very good temporal and spatial coincidence with some of the SDO reconnection events, bursts of 3–25 keV RHESSI X-rays, and weak interplanetary type IIIs detected by STEREO B. These radio, EUV, and X-ray observations provide strong evidence for the long-believed but unproven model that type III electrons are energized in or near reconnection regions, producing radio and X-ray emissions as they move away from and towards the Sun, respectively. The imperfect but significant correlations observed demonstrate that different special conditions must exist for strong radio and X-ray bursts to be produced by electrons from magnetic reconnection regions. These conditions are on the production and release of suitably energetic electrons (with additional auroral-style acceleration plausible for the X-ray emitting electrons) and on the production of observable radio (collective) and X-ray, EUV, and UV (single-particle) emissions from these electrons.

## Methods

### SDO images

SDO’s AIA instrument generates 4096 × 4096 pixel full-Sun images continuously on a 12 s cadence, with a pixel size of 0.6″ (or 435 km at the Sun) and effective spatial resolution of ≈ 1.6″, in 7 EUV and 3 white light/UV wavelength channels. The 94, 171, and 193 Å channels are centered^39^ on Fe XVIII, IX, and XII/XXIV lines corresponding to plasma temperatures near 6, 0.6, and 1.4/19 MK, respectively.

### Magnetic modeling

The Potential Field Source Surface (PFSS) model for the magnetic field (Fig. 2) was constructed using photospheric magnetic field data from SDO’s Helioseismic and Magnetic Imager (HMI)^51^ and the Solar Software (SSW) PFSS package. Schrijver and De Rosa^45^ detail the model assumptions and approach.

### Temperature and density modeling

We use a Differential Emission Measure (DEM) model to estimate the electron temperature T_e_ and number density n_e_ of the cusp structure in Figs 2 and 3. DEMs combine multi-wavelength observations with the associated instrumental temperature responses^52^ to determine the DEM as a function of temperature along the line of sight (LOS). Integrating the DEM over temperature for the range log T_e_ = 5.9–7.3 most appropriate for AIA^53^ yields the total emission measure (EM), which allows the density to be estimated using an assumed thickness over the LOS. We used the algorithim xrt_dem_iterative2, developed by Weber *et al*.^54^ and available through the SSW package, as implemented by Reeves *et al*.^55^. Using the 6 optically-thin, Fe-dominated AIA channels over the cusp structure in Fig. 3 (top left panel) results in a plot of DEM versus T_e_ (not shown). The total EM is 1.2 × 10^29^ cm^−5^. Weighting using the DEM and assuming a cusp thickness of half its width (5 pixels and so ≈ 2.2 Mm) yields <n_e_> = 2.3 × 10^10^ cm^−3^. The corresponding <T_e_> = 6.3 MK.

### Jet kinematics

Speeds are estimated along 47 linear slices that lie between the white arrows plotted in the lower-left and bottom panels of Fig. 2 and Movie B3, respectively. Intensities along each slice are background subtracted and binned together so that adjacent jets can be captured in one figure. Each column of the lower-right and top panels of Fig. 2 and Movie B3 represent the binned intensity at a single time, such that the horizontal stack illustrates the evolution of outflow material along the slice. The slope of each feature corresponds to its speed. Fitting lines to three of the most prominent jets, we find plane-of-sky speeds between ≈170 and 330 km s^−1^. Other studies of reconnection outflows have found similar values^55,56^.

## Electronic supplementary material

Supplementary Materials

Movie B1

Movie B2

Movie B3
