# Supplementary material for: Low Altitude Solar Magnetic Reconnection, Type III Solar Radio Bursts, and X-ray Emissions
Source: Sci Rep. 2018 Jan 26;8:1676. doi: 10.1038/s41598-018-19195-3 (PMC5786056; doi:10.1038/s41598-018-19195-3)
Supplement: Supplementary file 1 — Supplementary Materials [file 41598_2018_19195_MOESM1_ESM.pdf]

Version of 12 December 2017.

Supporting Materials for "**Low Altitude Solar Magnetic Reconnection, Type III Solar Radio Bursts, and X-ray Emissions**"

I.H. Cairns[1*], V.V. Lobzin[1,2], A. Donea[3], S.J. Tingay[4], P.I. McCauley[1], D. Oberoi[5], R.T. Duffin[1,4,6], M.J. Reiner[7,8], N. Hurley-Walker[4], N.A. Kudryavtseva[4,9], D.B. Melrose[1], J. C. Harding[1], G. Bernardi[10,11,12], J.D. Bowman[13], R.J. Cappallo[14], B.E. Corey[14], A. Deshpande[15], D. Emrich[4], R. Goeke[16], B.J. Hazelton[17], M. Johnston-Hollitt[18], D.L. Kaplan[19], J.C. Kasper[11], E. Kratzenberg[14], C.J. Lonsdale[14], M.J. Lynch[4], S.R. McWhirter[14], D.A. Mitchell [20,4], M.F. Morales[17], E. Morgan[16], S.M. Ord[4,11], T. Prabu[15], A. Roshi[21], N. Udaya Shankar[15], K.S. Srivani[16], R. Subrahmanyan[15,21], R.B. Wayth[4,22], M. Waterson[4,23], R.L. Webster[20,22], A.R. Whitney[14], A. Williams[4], and C.L. Williams[16]

[1] School of Physics, University of Sydney, Sydney, NSW 2006, Australia

* Correspondence to Prof. Iver Cairns at iver.cairns@sydney.edu.au

[2] Now at: Space Weather Services, Bureau of Meteorology, PO Box 1386, Sydney, NSW 1240, Australia

[3] Centre for Astrophysics, School of Mathematical Sciences, Monash University, Melbourne, VIC 3800, Australia

[4] International Centre for Radio Astronomy Research, Curtin University, Perth, WA 6845, Australia

[5] National Centre for Radio Astrophysics, Tata Institute for Fundamental Research, Ganeshkhind, Pune – 411007, India

[6] Department of Physics, Seattle University, Seattle, WA 98122-1090, USA

[7] The Catholic University of America, Washington, DC, USA

[8] NASA Goddard Space Flight Center, Greenbelt, MD 02330, USA

[9] Department of Cybernetics, Tallinn University of Technology, Tallinn 12 618, Estonia

[10] Square Kilometre Array South Africa (SKA SA), Cape Town, South Africa

[11] Harvard-Smithsonian Center for Astrophysics, Cambridge, USA

[12] Rhodes University, Grahamstown, South Africa

[13] Arizona State University, Tempe, USA

[14] MIT Haystack Observatory, Westford, MA 01886-1299, USA

[15] Raman Research Institute, Bangalore, India

[16] MIT Kavli Institute for Astrophysics and Space Research, Cambridge, USA

[17] University of Washington, Seattle, USA

[18] Victoria University of Wellington, Wellington, New Zealand

[19] University of Wisconsin--Milwaukee, Milwaukee, USA

[20] University of Melbourne, Melbourne, Australia

[21] National Radio Astronomy Observatory (NRAO), Charlottesville, USA

[22] ARC Centre of Excellence for All-sky Astrophysics (CAASTRO)

[23] Australian National University, Canberra, Australia

Supporting Material A: Active Regions and X-ray Flare Context: On 25 September 2011 the solar disk had the extended, complex, active region AR11302 with at least 3 large sunspots and an extensive magnetic loop system near the east equatorial limb. AR11296 was very near the northwest limb while an unnamed active region was near the equatorial disk center, both having large loop systems but no prominent sunspots. Multiple flares occurred in GOES X-ray data that day, including large M class events (fluxes > 10^{-6} $Wm^{-2}Hz^{-1}$) peaking near 02:30, 03:35, and 04:45 UT. Additional weak but detectable events peaked near 01:11:50, 01:13:20, and 01:19:50 UT, superposed on the decay phase of a C-class event just prior to 00 UT. Subtraction of this event's background leads to all three weak events having peak fluxes in the range for B-class events, although officially they are C-class events. See Figure 8 for the GOES X-ray data.

Supporting Material B: Detailed movies of the reconnection events: The existence and time sequence of the EUV brightenings and outflow events is clearest in movies of the SDO data, as presented here in Movies B1, B2, and B3. A brief summary is that two main bursts of activity occur 01:08 – 01:14 and 01:18 – 01:24 UT on 25 September 2011, with large outbursts near 01:13:00, 01:19:00-01:20:30, and 01:22:10 (± 12s due to the instrument cadence). Cusp-like features are discernible at least near 01:11:40, 01:19:00, and 01:21:20 UT in 171Å data, with brightenings near the cusps and along the current sheets. Loops and cusps develop at low altitudes, rise, and disappear; these changing magnetic topologies are expected for reconnection. Strong jets are directed outward and approximately southeast along open magnetic field lines from the upper portion of the current sheets near 01:13:00 – 01:14:20, 01:19:00 – 01:20:20, and 01:22:10 – 01:22:40. These form double-sided jet systems with more continuous westward-directed jets, which are directed downwards from the current sheets towards distinct and usually well-separated magnetic loop footpoints.

Movie B1. SDO-AIA (left) large-scale and (right) zoomed-in movies of the reconnection region in AR11302 and its evolving current sheets, loops, jets, open magnetic field lines, and associated outflows and downflows at 171 Å.

Movie B2. Zoomed-in, looping in forward and reverse, movie of SDO-AIA data at 171 Å for the time period 01:09:00 – 01:12:24 UT, emphasizing the downflows from the reconnection region but also showing the evolving current sheets, jets, open magnetic field lines, and outflows.

Movie B3. (Bottom) SDO-AIA image at 171 Å showing the reconnection region, an outflow along an open magnetic field line, and the analysis box (white lines) with central axis (thick white line) aligned approximately along the open magnetic field line. (Top) Distance-time kinematics of outflows, seen as intensifications in the 171 Å emission summed across the box for each distance along the central axis from the reconnection region, for the period 01:00 – 01:35 UT.

Supporting Material C: Interpretations of the special conditions for radio, EUV, UV, and X-ray emission from electrons leaving reconnection regions

One interpretation for these special conditions is that two different (but related) electron populations originating in reconnection regions produce type III bursts and nonthermal X-rays in distinct source regions: specifically, outward-going electrons produce type IIIs while downward-going electrons are accelerated by an additional mechanism as they approach the magnetic footpoints and produce X-rays. An analogy exists with Earth's magnetosphere, in which some energetic electrons leaving magnetotail reconnection sites reach auroral field lines and are accelerated downward by parallel electric fields and Alfven waves to produce auroral UV and X-ray emissions. Earth's reconnection outflows produce electron beams and Langmuir waves but appear to be radio-quiet. Another benefit of this interpretation is that the additional auroral electron acceleration may resolve the so-called "number problem" [15,19,36,37], which is that too many accelerated electrons are required to explain the observed nonthermal X-rays in terms of thick-target bremstrahlung from electrons accelerated in coronal reconnection regions. The low (~ 5-10 Mm) chromospheric heights of this paper's reconnection regions imply a much denser plasma than the corona and so reduces the number problem.

Another interpretation involves the very different emission mechanisms: the X-ray (and EUV) emissions are produced by single-particle collisional processes (primarily bremstrahlung) while type III bursts involve collective processes for the growth of Langmuir waves and their conversion into radio emission. These collisional and collective processes all depend on the number, energy, and distribution function of electrons leaving reconnection sites, leading to fluxes proportional linearly and nonlinearly, respectively, on the number of fast electrons. Specifically, the evolution of the Langmuir waves and radio emission involve development of an electron beam by time-of-flight effects from the acceleration site, growth of Langmuir waves via the electron beam instability and other processes, generation of radio waves via nonlinear Langmuir wave processes. Simulations show the physics to be strongly nonlinear, depending not just on the properties of the accelerated and background electrons [4,6,49,51] but also on variations in the electron and ion temperatures and density along the beam path[49,51].

Importantly, if the accelerated distribution in the reconnection site (location $x = 0$ to $2\Delta L$) is stable and has speeds $v_b \pm \Delta v_b$ then either an instantaneous release or a step-function increase to continuous outflow leads to a beam at a distance $L \gg \Delta L$ by time-of-flight effects for a time at most $2L/v_b\ (\Delta v_b/v_b + \Delta L / L)$. Thus larger acceleration regions with larger energy spreads lead to longer-lasting electron beams but at the cost of significantly larger instantaneous widths δv in velocity space. Longer release times also lead to larger δv. Smaller δv leads to enhanced type III emission since the growth rate for Langmuir waves is proportional to $(\delta v)^{-2}$ and the Langmuir waves grow with a smaller range of wavenumbers $\propto (\delta v)^{-1}$, thus increasing their energy density and so the nonlinear rates [4-6,49,51]. Thus larger type III emission is favoured for more rapid release events in smaller regions, balanced by the need for the source region to be large enough for the emission to be observable. However, a priori a larger acceleration region will produce more fast electrons and so bremstrahlung X-ray and EUV emission than a smaller source for otherwise identical electron and source conditions. Thus, the different dependences of the emission physics on electron beam properties provide several mechanisms by which differences in particle acceleration and release in reconnection events might produce the eight possible combinations of observable / unobservable EUV, X-ray, and type III radio emission. Moreover, the electron beam's evolution affects the X-ray emissions[18-21,], plausibly also true for the EUV/UV emissions. Finally, all three emissions have different intrinsic angular emission patterns and suffer different amounts of scattering and free-free absorption (due to their different wavelengths). In summary, the different growth (especially collective versus single-particle) and propagation physics make it very plausible that different special

conditions exist for production of observable radio, X-ray, and EUV emission from energetic electrons leaving solar magnetic reconnection regions.

Supporting Material D: The detailed acknowledgements for the MWA are as follows.

This scientific work makes use of the Murchison Radio-astronomy Observatory, operated by CSIRO. We acknowledge the Wajarri Yamatji people as the traditional owners of the Observatory site. Support for the MWA comes from the U.S. National Science Foundation (grants AST-0457585, PHY-0835713, CAREER-0847753, and AST-0908884), the Australian Research Council (LIEF grants LE0775621 and LE0882938), the U.S. Air Force Office of Scientific Research (grant FA9550-0510247), and the Centre for All-sky Astrophysics (an Australian Research Council Centre of Excellence funded by grant CE110001020). Support is also provided by the Smithsonian Astrophysical Observatory, the MIT School of Science, the Raman Research Institute, the Australian National University, and the Victoria University of Wellington (via grant MED-E1799 from the New Zealand Ministry of Economic Development and an IBM Shared University Research Grant). The Australian Federal government provides additional support via the Commonwealth Scientific and Industrial Research Organisation (CSIRO), National Collaborative Research Infrastructure Strategy, Education Investment Fund, and the Australia India Strategic Research Fund, and Astronomy Australia Limited, under contract to Curtin University. We acknowledge the Pawsey Supercomputing Centre, the iVEC Petabyte Data Store, the Initiative in Innovative Computing and the CUDA Center for Excellence sponsored by NVIDIA at Harvard University, and the International Centre for Radio Astronomy Research (ICRAR), a Joint Venture of Curtin University and The University of Western Australia, funded by the Western Australian State government.
